# HIGD1B, as a novel prognostic biomarker, is involved in regulating the tumor microenvironment and immune cell infiltration; its overexpression leads to poor prognosis in gastric cancer patients

**DOI:** 10.3389/fimmu.2024.1415148

**Published:** 2024-07-23

**Authors:** Shibo Wang, Siyi Zhang, Xiaoxuan Li, Xiangxue Li, Shufen Zhao, Jing Guo, Shasha Wang, Rui Wang, Mengqi Zhang, Wensheng Qiu

**Affiliations:** Department of Oncology, The Affiliated Hospital of Qingdao University, Qingdao, China

**Keywords:** HIGD1B, gastric cancer, prognostic biomarker, TME, immune infiltration, immunotherapy

## Abstract

**Background:**

HIGD1B (HIG1 Hypoxia Inducible Domain Family Member 1B) is a protein-coding gene linked to the occurrence and progression of various illnesses. However, its precise function in gastric cancer (GC) remains unclear.

**Methods:**

The expression of HIGD1B is determined through the TCGA and GEO databases and verified using experiments. The association between HIGD1B and GC patients’ prognosis was analyzed via the Kaplan-Meier (K-M) curve. Subsequently, the researchers utilized ROC curves to assess the diagnostic capacity of HIGD1B and employed COX analysis to investigate risk factors for GC. The differentially expressed genes (DEGs) were then subjected to functional enrichment analysis, and a nomogram was generated to forecast the survival outcome and probability of GC patients. Additionally, we evaluated the interaction between HIGD1B and the immune cell infiltration and predicted the susceptibility of GC patients to therapy.

**Results:**

HIGD1B is markedly elevated in GC tissue and cell lines, and patients with high HIGD1B expression have a poorer outcome. In addition, HIGD1B is related to distinct grades, stages, and T stages. The survival ROC curves of HIGD1B and nomogram for five years were 0.741 and 0.735, suggesting appropriate levels of diagnostic efficacy. According to Cox regression analysis, HIGD1B represents a separate risk factor for the prognosis of gastric cancer (p<0.01). GSEA analysis demonstrated that the HIGD1B is closely related to cancer formation and advanced pathways. Moreover, patients with high HIGD1B expression exhibited a higher level of Tumor-infiltration immune cells (TIICs) and were more likely to experience immune escape and drug resistance after chemotherapy and immunotherapy.

**Conclusion:**

This study explored the potential mechanisms and diagnostic and prognostic utility of HIGD1B in GC, as well as identified HIGD1B as a valuable biomarker and possible therapeutic target for GC.

## 1 Introduction

Gastric cancer (GC) is one of the most widespread and fatal diseases in the world. In 2020, there have been over 1 million new instances of GC worldwide, placing it fourth in terms of mortality among malignant tumors and fifth in terms of morbidity rate (1). In recent years, patients with GC have an improved outlook thanks to advancements in endoscopic and surgical procedures, as well as the application of adjuvant therapies such as chemotherapy, targeted therapy, and immunotherapy (2, 3). Nonetheless, due to the substantial molecular and phenotypic heterogeneity of GC (4), most patients with advanced gastric cancer still have a dismal prognosis, with a 5-year survival rate of less than 30% (5, 6). Therefore, searching for new, highly sensitive, and specific biomarkers and therapeutic targets is imperative to improve the present treatment approaches for GC.

Hypoxia is one of the crucial stress modes that cause cell damage and even death (7), which is intimately linked to conditions including cancer, heart disease, and stroke (8). It aids in the reconstruction of the tumor microenvironment (TME) and facilitates the growth and metastasis of malignancies. The HIG1 hypoxia inducible domain (HIGD) gene family is a putative anti-apoptotic factor since it is elevated during hypoxia and can influence several critical biological processes (9, 10). For instance, in hypoxic settings, hypoxia-inducible factor 1α (HIF-1α) induces HIGD1A expression, which exerts anti-apoptotic properties by blocking the release of cytochrome C (Cc) and diminishing caspase activity (11–13). In addition, by controlling AMPK activity and cellular reactive oxygen species (ROS) levels in the body, HIGD1A can lessen tumor cell death and contribute to the development and spread of malignancy (14).

HIGD1B is a significant member of the HIGD family, with the HIGD1B genome located on chromosome 17q21.31. The gene encodes the protein HIG2A, composed of 99 amino acids and abundantly expressed in the brain, heart, lung, and subcutaneous adipose tissue (9, 15). Other homologous proteins include HIGD-1A, -1C, -2A, and -2B. With more than 40% homology, HIGD-1B and HIGD-1A are extremely analogous in the transmembrane domain (15). Studies have proved that by postponing the cleavage of OPA1, HIGD1B can inhibit hypoxia or CCCP-induced mitochondrial rupture and cell death. Its mechanism of governing mitochondrial fusion is similar to HIGD1A, and knocking down HIGD-1B can accelerate apoptosis of myocardial cells in hypoxic surroundings (16). Furthermore, HIGD1B is involved in the onset and advancement of intracranial aneurysms (IA), growth hormone-secreting pituitary adenomas (GHomas), and lung cancer (17–19). Nevertheless, little is known about the expression and mechanism of HIGD1B in GC, and its diagnostic and prognostic value in GC is not fully understood.

In this article, we analyzed the expression of HIGD1B in GC and normal gastric tissues by multiple independent cohorts from public databases, and we verified our findings with cell experiments. We accessed the possible roles of HIGD1B in the genesis and progression of GC through various enrichment analysis methods. Researchers then explored the relationship between HIGD1B and clinicopathological elements, TME, and immune cell infiltration of GC, thoroughly and systematically evaluated the diagnostic and prognostic value of HIGD1B in GC, predicted the effectiveness of chemotherapy and immunotherapy, and ultimately identified HIGD1B as a novel prognostic biomarker for GC.

## 2 Materials and methods

### 2.1 Data collection

Transcriptome information (TPM) and matching clinical data of gastric cancer and adjacent tissues downloaded from the Cancer Genome Atlas (TCGA) (https://portal.gdc.cancer.gov/) and Gene Expression Comprehensive (GEO, https://www.ncbi.nlm.nih.gov/) databases (**Supplementary Table S1**). The TCGA-STAD cohort has 36 normal and 410 cancer specimens; 439 of these samples provide prognostic data. The GSE29272 queue represents 134 normal and 134 cancer samples. The GSE54129 queue includes 21 normal and 111 cancer samples. There are 433 and 300 gastric cancer patients and their prognosis information in the GSE84437 and GSE62254 queues, respectively. HIGD1B expression is validated using GSE29272 and GSE54129. GSE84437 and GSE62254 were used for forecasting outcome. The Cancer Immunohistochemical Atlas (TCIA, https://tcia.at/patients) provides information on immunotherapy. Somatic mutation data are derived from UCSC Xena database (https://xenabrowser.net/datapages/).

### 2.2 Cell culture

Both human gastric normal cell (GES-1) and GC cell (AGS, HGC-27) lines were offered by the Chinese Academy of Sciences Cell Bank (Shanghai, China). These cell lines were identified by STR and tested negative for mycoplasma. These cell lines were cultured in Losvi-Parker Memorial Institute (RPMI)-1640 medium (Corning, USA) supplemented with 10% fetal bovine serum (FBS) (Corning, USA), 100 U/mL penicillin (Corning, USA), and 100 μg/mL streptomycin (Corning, USA) at 37°C, 5% CO2.

### 2.3 Quantitative real-time polymerase chain reaction

Extract total RNA from cells using TRIzol reagent and synthesize cDNA using reverse transcription kit (TermoFisher Scientific, USA) as instructed. In the qRT-PCR experimental process, 2 µl of reverse transcription product, 7.2 µl of DEPC, 10 µl of SYBR, and 0.4 µl of forward and reverse primer were utilized. Perform qRT-PCR reaction under the following conditions: pre-denaturation 95°C 30 seconds, followed by a cycle (Reps: 40) of 95°C 10 seconds, 60°C 30 seconds. Finally, draw the PCR product’s melting curve at 95°C for 15 seconds, 60°C for 60 seconds, and 95°C for 15 seconds (20). The primer sequences are as follows:

HIGD1B-F(5′-GTACCACCTGACGACGAAGACTG-3′).

HIGD1B-R(5′-ATCCTGTATGCTGCTACCACCAAG-3′).

GAPDH-F(5’-TGCACCACCAACTGCTTAGC-3’).

GAPDH-R (5’-GGCA TGGACTGTGGTCATGAG-3’).

GAPDH as an internal reference, employing the 2^−ΔΔCt^ method to determine the relative expression level of HIGD1B. The experiment was repeated three times.

### 2.4 Western blot

After washing the cultivated cells with PBS, the total protein was extracted using RIPA buffer (KWB002; KIGENE Biotech, China). Collect the supernatant after centrifugation at 4°C for 10 minutes and measure the protein concentration using the BCA assay kit (KWB011, KIGENE Biotech, China). The protein was transferred to the PVDF membrane (KWB047; KIGENE Biotech, China) after being separated on a 10% SDS-PAGE gel. Incubate the membrane at room temperature in a 5% skim milk solution for 1-2 hours, and then primary antibodies anti-HIGD1B (ABIN2175800, antibodies-online, China; 1:1000) and β-actin (KWB040-R; KIGENE, China; 1:1000) was incubated overnight at 4°C. After that, wash the membrane in TBST for 30 minutes and leave it in the secondary antibody conjugated with HRP at 37°C for 1 hour. After TBST washing again, visualize protein bands using ECL assay kits (KWB032; KIGENE Biotech, China).

### 2.5 The relationship between HIGD1B and clinicopathological characteristics of GC

Researchers generated high and low expression groups for individuals in the TCGA-STAD, GSE62254, and GSE84437 cohorts based on the median expression of HIGD1B and derived survival curves employing Kaplan-Meier analysis (21). In the TCGA-STAD dataset, we studied the association between HIGD1B and clinical pathological indicators, as well as the correlation between HIGD1B and GC prognosis among various clinical subgroups. The accuracy of HIGD1B in predicting survival time and survival rate in GC patients was then assessed using receiver operating characteristic (ROC) curves (22). Further, using Cox analysis, the expression of HIGD1B and other clinical features (such as age, gender, cancer grade, and stage) were elucidated concerning the overall survival of GC patients (23).

### 2.6 Differential analysis and functional enrichment analysis

Initially, we utilized the expression of HIGD1B to categorize GC patients in the TCGA cohort into high and low-expression groups and implemented the “limma” package (| LogFC |>1 and FDR<0.05) to seek out differentially expressed genes (DEGs) between the two groups (24). Next, using the “enrichplot” and “clusterProfiler” packages, GO and KEGG enrichment analyses were undertaken to discover biological processes and signaling pathways linked to HIGD1B (q-value<0.05) (25). Likewise, Gene Set Enrichment Analysis (GSEA) was also applied to clarify the possible mechanisms and pathways of HIGD1B in GC. The selected reference molecular database was “c2. cp. Kegg. Hs. symbols. gmt”, and | NES |>1 and p-value<0.05 were regarded as significantly enriched (26).

### 2.7 Establish and assess a nomogram

To better align with clinical practice, researchers designed a nomogram (27) through the “rms” and “survival” packages (28) based on all independent prognostic risk factors determined by Cox regression analysis to measure the survival time and survival rate of GC patients. Then, ROC curves were used to compare the nomogram’s prediction power with other clinicopathological parameters, and the dependability of the nomogram was evaluated by calibrating the curve.

### 2.8 Analysis of tumor microenvironment and immune cell infiltration

We compute the corresponding scores by employing the ESTIMATE methodology (29) to assess the fraction of immune, stromal, and tumor cells in the tumor microenvironment of gastric individuals with cancer. For patients in the high and low HIGD1B expression groups, the infiltration proportion and abundance of TIICs were evaluated via the CIBERSORT and single sample gene set enrichment analysis (ssGSEA) methods (30–32). Additionally, the correlation between HIGD1B expression and certain TIICs was examined using Spearman analysis.

### 2.9 Prediction of immunotherapy and drug sensitivity

We measured each GC sample’s Dysfunction, Exclusion, and TIDE scores through the TIDE website (http://tide.dfci.harvard.edu/), and the connection between HIGD1B and MSI-related indicators was analyzed. Subsequently, the tumor mutation burden (TMB) score was determined from somatic mutation data, and a waterfall plot presented the somatic mutation landscape. The prognostic differences between several TMB groups were evaluated using K-M curves. To forecast the clinical effectiveness of immunotherapy in patients with GC, we also examined the relation between HIGD1B and CTLA-4 in conjunction with PD-1 immunotherapy. Standard chemotherapeutic medicines were checked for sensitivity utilizing the “oncoPredict” package (33). Drug sensitivity was demonstrated in relation to the semi-inhibitory concentration value.

### 2.10 Statistical analysis

R software (version 4.2.2) was applied for bioinformatics statistics and plotting. The “timeROC” and “survival” packages of R were employed for the ROC curve and Cox regression analysis, respectively. Wilcoxon test was utilized for intergroup analysis. Kaplan-Meier curve was implemented for survival analysis, and Spearman was used for correlation analysis. The experimental data was analyzed using GraphPad Prism (version 9.3.1), and a one-way analysis of variance (ANOVA) was used to compare the relative expression levels of HIGD1B. Statistics are deemed significant when p<0.05. *P<0.05; **, P<0.01; ***, P<0.001; ****, P<0.0001.

## 3 Results

### 3.1 Expression of HIGD1B in pan-cancer and gastric cancer

**Figure 1:**
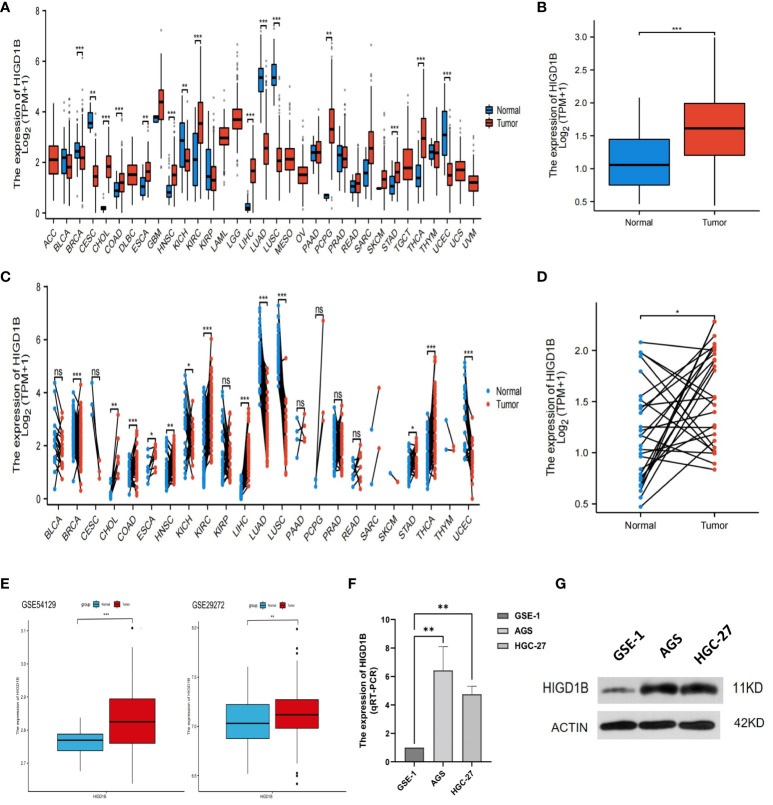
Analyzing and validating the expression of HIGD1B in pan-cancer and gastric cancer. **(A)** Expression of HIGD1B in pan-cancer non-paired samples. **(B)** Expression of HIGD1B in GC and adjacent tissues (non-paired) of the TCGA-STAD cohort. **(C)** Expression of HIGD1B in pan-cancer paired samples. **(D)** Expression of HIGD1B in GC and paired adjacent tissues of the TCGA-STAD cohort. **(E)** Expression of HIGD1B in GC and adjacent tissues in the GSE54129 and GSE29272 cohorts. **(F)** Detection of HIGD1B expression in gastric epithelial cells (GSE-1) and GC cell lines (AGS and HGC-27) by the qRT-PCT assay. **(G)** Detection of HIGD1B expression in gastric epithelial cells (GSE-1) and GC cell lines (AGS and HGC-27) by Western blot assay. *P < 0.05; **P < 0.01; ***P < 0.001. TCGA, The Cancer Genome Atlas; STAD, stomach adenocarcinoma; GC, gastric cancer.

According to the analysis of pan-cancer data downloaded from the TCGA database (**Supplementary Table S2**), HIGD1B was observed to be significantly upregulated in the tumor tissues of COAD, ESCA, HNSC, KIRC, LIHC, STAD, and THCA, but lowered in BRCA, KICH, LUAD, LUSC, and UCEC (**Figures 1A, C**). In the TCGA-STAD cohort, HIGD1B was discovered to be considerably higher in gastric cancer tissue as compared to normal gastric tissue (p<0.001) (**Figure 1B**). Likewise, it was noticed that gastric cancer tissue had a higher expression of HIGD1B in comparison to 27 paired adjacent tissues (p<0.05) (**Figure 1D**). Then, we downloaded the GSE29272 (containing 134 GC and 134 adjacent samples) and GSE54129 (containing 111 GC and 21 adjacent samples) cohorts from the GEO database for analysis to further examine the expression of HIGD1B in GC and adjacent tissues. The findings confirmed that HIGD1B in gastric cancer tissue was considerably greater (p<0.01) than adjacent tissue in both cohorts (**Figure 1E**). Additionally, researchers implemented qRT-PCR and Western blot assays to determine the expression of HIGD1B mRNA and protein in GC cell lines (**Supplementary Table S3**), suggesting the expression of HIGD1B in HGC-27 and AGS cells was substantially greater than GSE-1 (**Figures 1F, G**).

### 3.2 The relationship between HIGD1B and clinical pathological characteristics of gastric cancer

**Figure 2:**
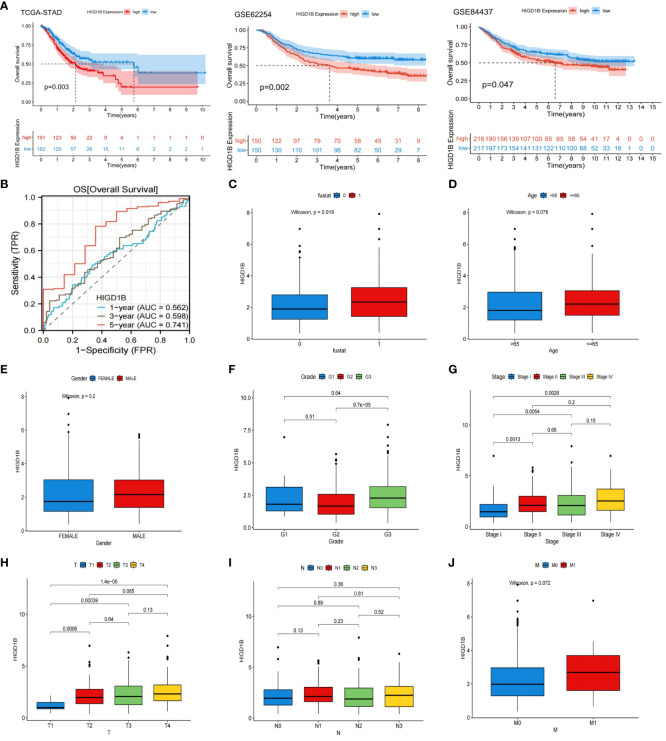
Systematic evaluation the relationship between the HIGD1B and clinicopathological features. **(A)** Kaplan-Meier curves of high and low HIGD1B expression subgroups in the TCGA-STAD, GSE62254 and GSE84437 queues. **(B)** ROC curve of HIGD1B for predicting 1, 3, and 5-year survival in the TCGA-STAD queue. **(C)** The expression levels of HIGD1B in the surviving (fustat=0) and deceased (fustat=1) populations. **(D)** Expression of HIGD1B in age subgroups. **(E)** Expression of HIGD1B in gender subgroups. **(F)** The expression of HIGD1B in different pathological grading populations. **(G)** The expression of HIGD1B in staging subgroups. **(H–J)** The expression of HIGD1B in T stage, N stage, and M stage subgroups. TCGA, The Cancer Genome Atlas; STAD, stomach adenocarcinoma; ROC; receiver operating characteristic; GC, gastric cancer.

**Figure 3:**
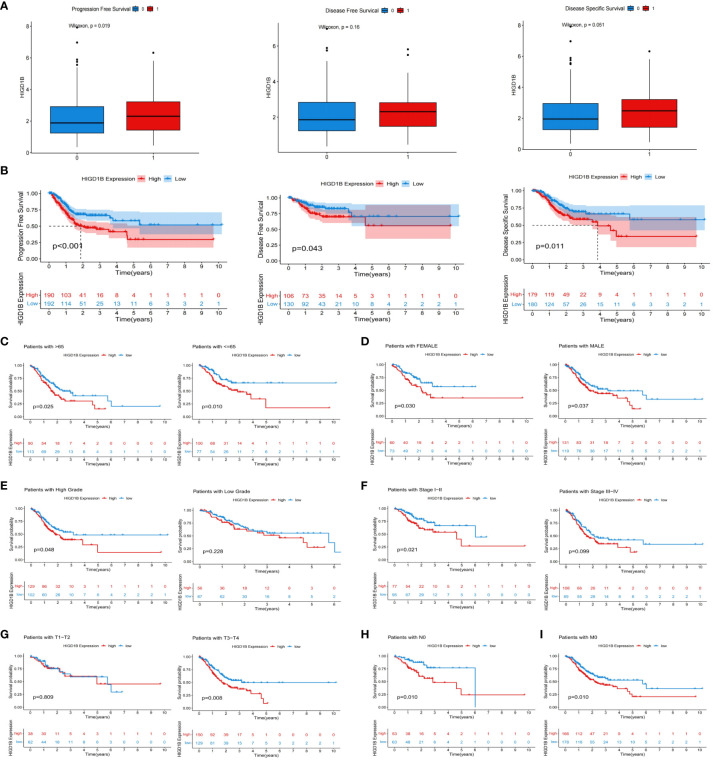
The relationship between HIGD1B and the prognosis of GC. **(A)** The relationship between HIGD1B’s expression and PFS, DFS, and DSS. **(B)** K-M curves of PFS, DFS, and DSS in the high and low HIGD1B expression subgroups. **(C–G)** The K-M curve of OS between different HIGD1B groups based on age, gender, pathological grading, stage, T-stage stratification. **(H)** The K-M curve between different HIGD1B subgroups in N0 population. **(I)** The K-M curve between different HIGD1B subgroups in M0 population. GC, gastric cancer; PFS, Progression Free Survival; DFS, Disease Free Survival; DSS, Disease Free Survival; K-M Kaplan-Meier; OS, overall survival.

In the TCGA-STAD, GSE65524, and GSE84437 cohorts, all patients were classified into high and low groups based on the median expression of HIGD1B, respectively. K-M curves were applied to investigate the association between HIGD1B and overall survival (OS), results revealed that patients with high expression of HIGD1B had shorter survival time in all cohorts (p<0.05) (**Figure 2A**). To gauge the diagnostic worth of HIGD1B, we set up receiver operating characteristic (ROC) curves using GC patients from the TCGA database. The AUC values for 1, 3, and 5-year survival rates were 0.562, 0.598, and 0.741, respectively, indicating that the diagnostic efficacy of HIGD1B in predicting GC survival is appropriate (**Figure 2B**). In the TCGA-STAD cohort, HIGD1B expression was greater in the population reaching PFS (p<0.05), while there was no significant difference in DFS and DSS (**Figure 3A**). In addition, patients with GC who expressed high levels of HIGD1B also showed shorter PFS, DFS, and DSS (**Figure 3B**), implying a worse prognosis for this population. The correlation between HIGD1B and the clinicopathological parameters of gastric cancer was then evaluated, and it was discovered that there was no statistically significant variance in HIGD1B expression among age, gender, N and M staging populations (**Figures 2D, E, I, J**), but that there was higher expression of HIGD1B in the death population (fustat=1), higher pathological grade (G3), later stage and T stage groups (**Figures 2C, F–H**). Further, based on clinical-pathological feature stratification, we investigated the prediction capacity of HIGD1B for OS in GC patients. K-M analysis revealed that in terms of age (>65/<=65), gender (male and female), high grade, Stage I-II, T3-T4, N0, and M0 subgroups, patients with low HIGD1B expression had a considerably better prognosis than those with high HIGD1B expression (**Figures 3C–I**).

### 3.3 The potential mechanism of HIGD1B affecting gastric cancer

**Figure 4:**
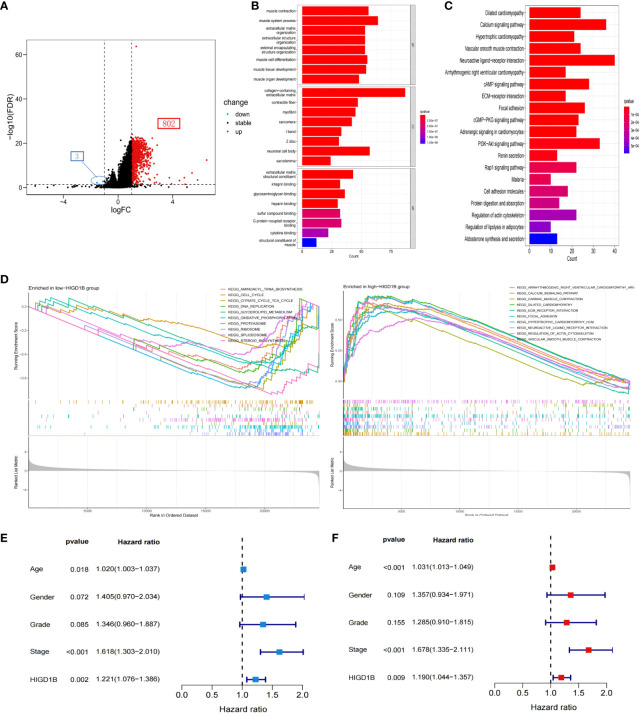
Functional enrichment analysis and Cox regression analysis. **(A)** Volcano maps of all DEGs between high and low HIGD1B expression groups. **(B)** GO analysis of DEGs between high and low HIGD1B subgroups. **(C)** KEGG analysis of DEGs between high and low HIGD1B subgroups. **(D)** GSEA analysis of the primary enriched pathways in high and low HIGD1B groups. **(E)** Univariate Cox regression analysis of HIGD1B and clinical parameters in the TCGA cohort. **(F)** Multivariate Cox regression analysis of HIGD1B and clinical parameters. DEGs, differentially expressed genes; GO, Gene Ontology; KEGG, Kyoto Encyclopedia of Genes and Genomes; GSEA, gene set enrichment analysis.

Initially, the TCGA-STAD cohort’s GC patients were split into groups with high and low expression, and the differential expression genes (DEGs) between the two groups were identified (3 down-regulated and 802 up-regulated) (**Figure 4A**; **Supplementary Table S4**). The possible processes and pathways of HIGD1B were then explored by doing GO and KEGG analyses on these genes (**Supplementary Table S5**). Among them, GO analysis uncovered that these DEGs mainly involve biological processes and molecular functions like “muscle system,” “integral binding,” and “extracellular matrix and structure organization” (**Figure 4B**). The KEGG analysis indicated that these DEGs were enriched in cell-matrix pathways such as “Cell adhesion molecules” and “ECM receptor interaction,” as well as cell signaling pathways such as “cAMP,” “cGMP-PKG,” “Rap1”, and “PI3K-Akt” (**Figure 4C**), which are closely connected to the occurrence and development of hypoxia, inflammation, and cancer (34–37). Additionally, the researchers employed GSEA analysis to examine the functional distinctions between the groups with different HIGD1B expressions. As per the research findings, the low HIGD1B group is linked to cellular metabolic processes like “Cell cycle,” “DNA replication,” “Ribosome,” and “Oxidative phosphorylation. “ Conversely, the high HIGD1B group’s pathways are obviously linked to “Calcium,” “Hedgehog,” 、 “TGF-β,” 、 “Wnt,” and “Focal adhesion” signaling paths (**Figure 4D**; **Supplementary Table S6**). Thus, we speculate that these tumors and stromal signaling pathways are connected to the poor prognosis of patients with elevated HIGD1B.

### 3.4 Construct and evaluate a clinical nomogram

**Figure 5:**
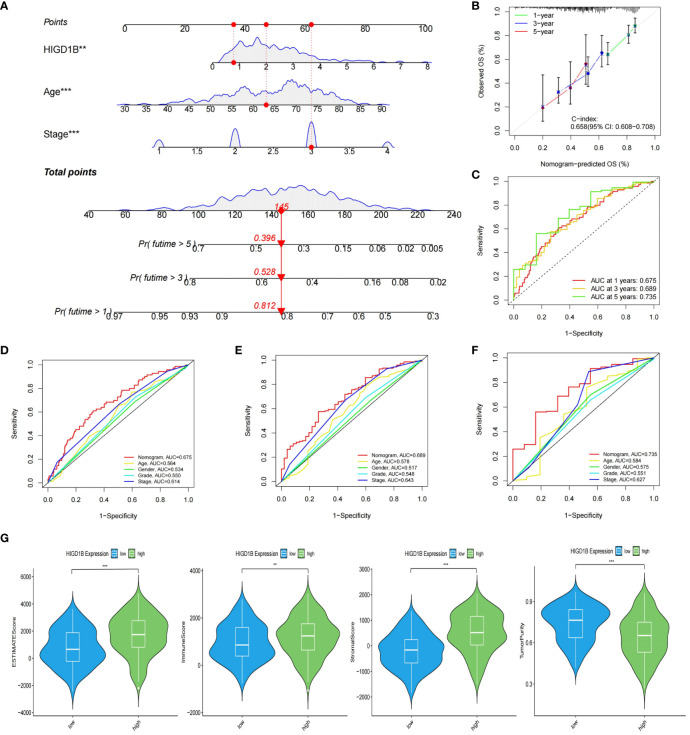
Construction of nomogram and evaluation of TME. **(A)** The nomogram created based on the HIGD1B, Age and Stage. **(B)** Calibration plots for nomograms at 1, 3, and 5-years. **(C)** ROC curve of the nomogram for predicting 1, 3, and 5-year survival. **(D–F)** ROC curve for predicting 1, 3, and 5-year survival according to the nomogram and other clinical features. **(G)** The proportion of stromal, immune, and tumor cells in the TME. **P < 0.01; ***P < 0.001. ROC, receiver operating characteristic; TME, tumor microenvironment.

To investigate the potential of HIGD1B as an independent predictor, we first performed a univariate Cox analysis and found that age (HR=1.020, p=0.018), stage (HR=1.618, p<0.001), and HIGD1B (HR=1.221, p=0.002) were significantly correlated with prognosis (**Figure 4E**). Multivariate Cox analysis exhibited that age (HR=1.031, p<0.001), stage (HR=1.678, p<0.001), and HIGD1B (HR=1.190, p<0.009) can independently predict the outcome of GC patients (**Figure 4F**; **Supplementary Table S7**). Subsequently, we produced a nomogram using parameters with p<0.05 from Cox analysis to further enhance clinical practicality. **Figure 5A** indicated that the nomogram predicts the 1, 3, and 5-year survival rates of a patient in the TCGA-STAD cohort to be 0.812, 0.528, and 0.396, respectively. The calibration curve (C-index: 0.658) illustrated the consistent capacity for prediction of the nomogram (**Figure 5B**). Moreover, the AUC values of the ROC curves for the 1, 3, and 5-year survival rates in the nomogram were 0.675, 0.689, and 0.735, respectively (**Figure 5C**), and they were superior to conventional clinical features in predicting the prognosis of GC patients (**Figures 5D–F**). In summary, we have demonstrated the efficiency and precision of the nomogram from various perspectives.

### 3.5 The relationship between HIGD1B and immune cell infiltration

**Figure 6:**
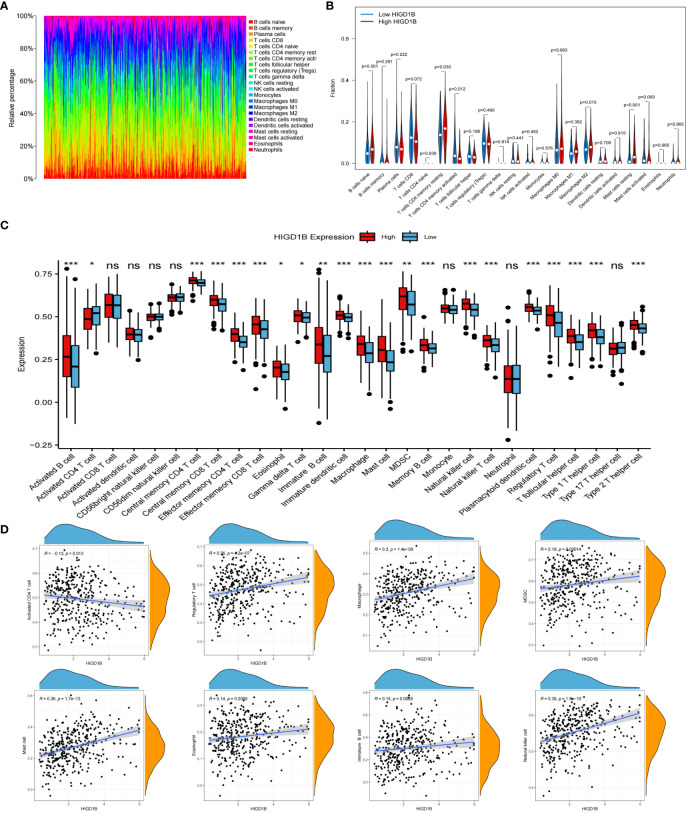
Immune cell infiltration analysis. **(A)** Evaluating the proportion of 22 types of TIICs employing the CIBERSORT algorithm. **(B)** Expression levels of 22 TIICs in high and low HIGD1B expression groups. **(C)** Examining the infiltration of TIICs in high and low HIGD1B groups using the ssGSEA algorithm. **(D)** Spearman analysis between HIGD1B expression and several TIICs (including Activated CD4 T cell, Regulatory T cell, Macrophage cell, MDSC and so on). ns P>0.05; *P < 0.05; **P < 0.01; ***P < 0.001. TIICs, tumor-infiltrating immune cells; ssGSEA, single-sample gene set enrichment analysis.

Tumor microenvironment (TME) is known for its immunosuppression and induction of drug resistance (38, 39), which can promote tumor cell proliferation and invasion, thereby adversely influencing the prognosis (40, 41). In addition, tumor-infiltrating immune cells (TIICs) are an important component of the tumor microenvironment, and an ever-growing body of reports have confirmed that TIICs are involved in cancer progression and recurrence (42–45), both TME and TIICs are crucial to the initiation and development of cancer. We first employed the ESTIMATE method to determine the proportion of tumor, stromal, and immune cells in the TME of GC patients. The findings implied that the high HIGD1B group had higher stromal, immune, and estimated scores, while the tumor purity score was lower (**Figure 5G**). Subsequently, using the CIBERSORT technique, the researchers assessed the percentage of all sample TIICs in the TCGA cohort (**Figures 6A, B**). The low HIGD1B group showed higher infiltration of T cells CD4 memory activated B with anti-tumor effects (46), while the high HIGD1B group had more macrophage M2 infiltration. Studies have indicated that it is associated with high expression of TGF-β, IL-4, IL-13, and IL-10, suppressing the inflammatory response and encouraging tumor angiogenesis and distant metastasis (47, 48). Furthermore, we investigated the association between TIICs and HIGD1B using ssGSEA and discovered that the majority of immunosuppressive cells were highly infiltrated in the high HIGD1B group (**Figure 6C**). Spearman analysis revealed HIGD1B has a negative correlation with activated CD4T cells but a positive correlation with T-Reg, MDSC, and Mast cells (**Figure 6D**). According to these findings, HIGD1B may regulate the infiltration and differentiation of TIICs to form highly inhibitory TME, thereby inhibiting immune response, promoting immune escape, and worsening the prognosis for patients with gastric cancer. All immune infiltration related data are in **Supplementary Table S8**.

### 3.6 Prediction of immunotherapy efficacy

**Figure 7:**
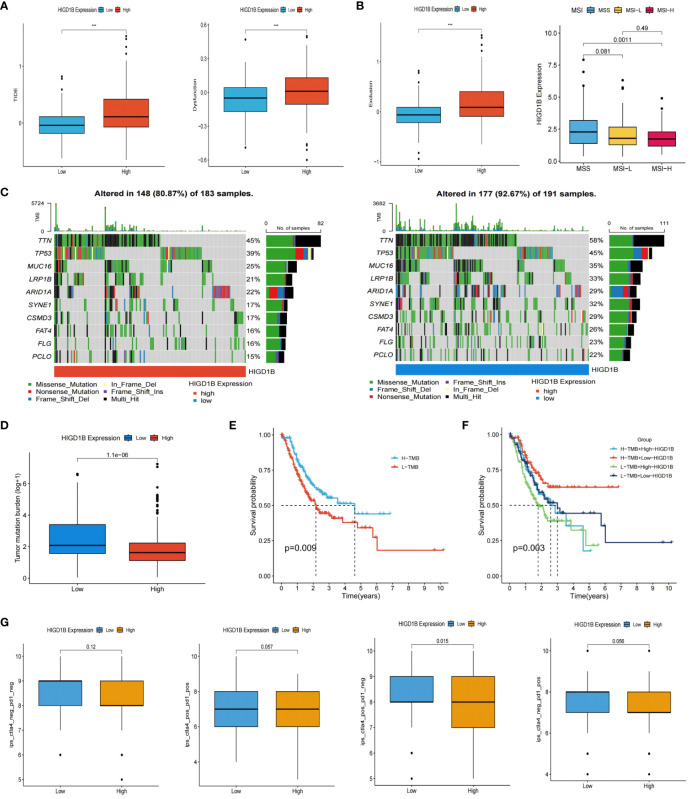
Prediction of immunotherapy for GC. **(A)** The scores TIDE, dysfunction, and exclusion in high and low HIGD1B groups. **(B)** Analysis of HIGD1B and microsatellite state (MSI). **(C)** Waterfall plotting of somatic mutations. **(D)** TMB levels in high and low HIGD1B expression groups. **(E)** Kaplan-Meier curve of OS in high and low-TMB groups. **(F)** Kaplan-Meier curve show different survival among the four groups that combined TMB with HIGD1B. **(G)** Analysis of the combined application of anti-PD-1 and anti-CTLA-4 antibodies in distinct HIGD1B groups. ***P < 0.001. GC gastric cancer; TIDE, tumor immune dysfunction and exclusion; TMB, tumor mutational burden; OS, overall survival.

**Figure 8:**
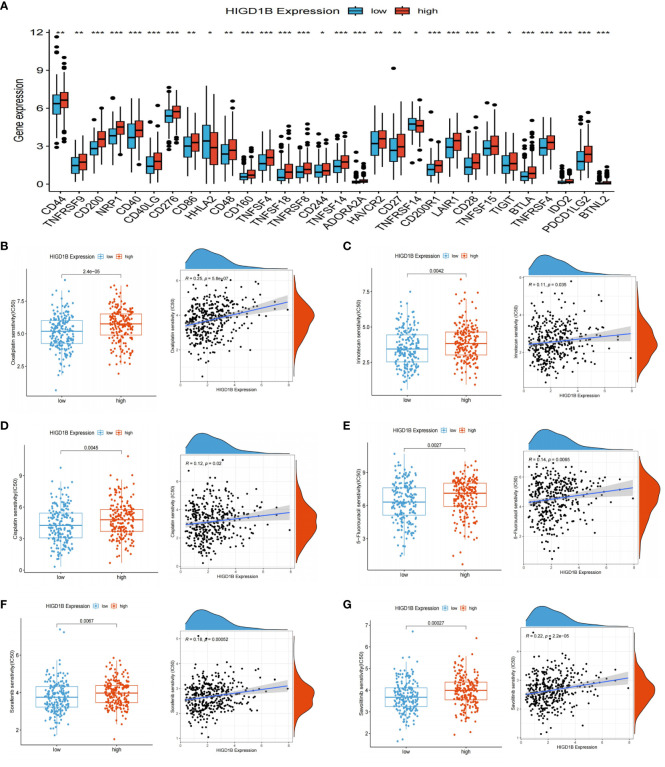
ICIS and drug sensitivity analyses. **(A)** Expression of ICIs in high and low HIGD1B expression groups. **(B–E)** Sensitivity analysis of chemotherapy drugs used for standard treatment of gastric cancer in clinical practice. Differences in sensitivity to chemotherapy drugs (including Oxaliplatin, Irinotecan, Cisplatin, 5-fluorouracil) among different subgroups of HIGD1B, and correlation between chemotherapy drugs IC50 value and HIGD1B expression. **(F, G)** Sensitivity analysis of targeted drugs (like Sorafenib and Savolitinib) in populations with high and low expression of HIGD1B. *P < 0.05; **P < 0.01; ***P < 0.001. ICIs, immune checkpoint inhibitors.

As immunotherapy continues to advance, cancer patients’ survival times have extended, and their quality of life has improved substantially compared to before, demonstrating its enormous application prospects in tumor treatment (49, 50). However, not all cancer populations are sensitive to immunotherapy because of individual variances. Thus, we need to identify more targets to expand the options for immunotherapy. The researchers computed the TIDE score of GC patients in the TCGA dataset and investigated its connection with HIGD1B (**Supplementary Table S9**). They found that the high HIGD1B group exhibited higher TIDE, exclusion, and dysfunction scores (**Figure 7A**), which means that the high HIGD1B group may be more prone to immune escape and less responsive to immunotherapy (51). In addition, Microsatellite instability (MSI)/DNA mismatch repair (MMR) is of great significance for the diagnosis, prognosis assessment, and treatment selection of various malignancies (52), especially digestive tract tumors such as gastric cancer and colorectal cancer (53, 54). **Figure 7B** illustrates that the HIGD1B expression in the MSI-H subgroup is considerably lower (p<0.01) in comparison to the MSS subgroup, indicating that patients with low HIGD1B expression have a greater chance of receiving immunotherapy. Research has indicated that most cancer mutations are somatic mutations, and approximately 90% of oncogenes exhibit somatic mutations, such as TP53 and TERT gene mutations that frequently occur in cancer lineages. These mutations also have a significant role in developing treatment strategies for tumors (55). We downloaded the TCGA-STAD queue’s somatic mutation data from the UCSC website for analysis. The waterfall plot uncovered that the mutation incidence was higher in the group with low HIGD1B expression (92.67% vs. 80.87%), with the most common type of mutation being missense mutations. The three genes with the highest prevalence of mutations were TTN, TP53, and MUC16 (**Figure 7C**). We then calculated the TMB scores of each GC patient. As shown in **Figures 7D, E**, the TMB value was significantly higher (p<0.001) in the low-HIGD1B expression group, and patients in the H-TMB subgroup had a longer survival time (p<0.01). The population in the H-TMB+L-HIGD1B group had the most excellent prognosis in the combined study of TMB and HIGD1B (**Figure 7F**). Immunotherapy provides a new approach to tumor treatment with unique advantages and enormous potential. Immune checkpoint inhibitors (ICIs) are a vital component of immunotherapy (56), and we forecast the immune response of GC by examining ICIs (**Figure 8A**). In addition, ICIs (PD-1 combined with CTLA-4) demonstrated that the PD-1 positive combined with CTLA-4 negative treatment group showed superior efficacy in the population with low HIGD1B expression, whereas there was no significant difference in the other three groups (**Figure 7G**).

### 3.7 Drug sensitivity analysis

Drug sensitivity analysis revealed that the semi-inhibitory concentrations of several clinically standard first—or second-line chemotherapy drugs (including oxaliplatin, 5-fluorouracil, cisplatin, and irinotecan) and targeted drugs are positively correlated with HIGD1B expression (**Figures 8B–G**). This indicates that individuals with low HIGD1B expression are more responsive to these drugs and have a higher likelihood of benefiting from them.

## 4 Discussion

Gastric cancer is one of the malignant tumors with the highest incidence rate in the world. Most patients are in the advanced stage when they are diagnosed. At present, only chemotherapy, targeted drugs (like trastuzumab), and some immune checkpoint inhibitors (such as nivolumab and pembrolizumab) are available in clinical practice, and the prognosis is poor. Therefore, exploring novel biomarkers has excellent prospects for early detection of gastric cancer, prognostic assessment, and prediction of therapeutic efficacy.

The relationship between hypoxia and tumor is inseparable, one of the main characteristics of cancer is hypoxia (57). Cancer cells have traits such as vigorous metabolism, rapid proliferation, and high energy demand. A hypoxic environment forms when there is a more significant requirement for oxygen than there is supply, which causes metabolic alterations. On the one hand, it induces neovascularization by stimulating cells to release erythropoietin (EPO) and angiogenic factors (58–60). On the other hand, it promotes the activation and proliferation of stromal cells, reshapes the tumor microenvironment, and exacerbates tissue hypoxia (61). These will help the tumor progress and make the patient more resistant to treatment. In addition, hypoxia can also generate a lot of reactive oxygen species (ROS), harm healthy cell’s DNA, increase the frequency of gene mutation, and ultimately cause cancer (62). Pursuant to current research, the HIGD gene family is induced expression by hypoxia-inducible factor-1α (HIF-1α) in hypoxic conditions, participates in the assembly of mitochondrial complexes, and regulates mitochondrial homeostasis, affect a range of physiological and pathological processes, and be a significant factor in numerous illnesses (particularly cardiovascular diseases, diabetes, and cancer). It is worth noting that HIF is a transcription factor extensively distributed in the human body during hypoxia. The activation of HIF, which contributes to the metabolic reprogramming of tumor cells (like the renowned Warburg effect) and supports the formation of an immunosuppressive microenvironment (by inhibiting CTLs and recruiting Tregs), is one of the primary mechanisms by which tumor cells can survive in hypoxic environments (63). Moreover, HIF is modulated by multiple paths, including PI3K-mTOR, JAK-STAT3, and Notch signaling pathways (64–66). Its overexpression is intimately linked to the growth, metastasis, and recurrence of cancer and may lead to tumor resistance to chemotherapy and immunotherapy.

The HIGD family includes HIGD1A, -1B, -1C, -2A, and -2B. The most studied gene is HIGD1A, a mitochondrial inner membrane protein that plays a crucial role in regulating cellular metabolic homeostasis and anti-apoptosis (67, 68). HIGD1A has a dual effect of promoting and inhibiting cancer and is regarded as HIF-1α’s target genes. HIGD1A weakens oxidative stress during hypoxia and glucose deficiency by activating the AMPK pathway, inhibiting mitochondrial respiration, reducing ROS generation, and mediating cell dormancy, alleviating tumor cell death (14, 15, 69). HIGD1A has been proven to be a meaningful biomarker in pancreatic cancer and glioma (70, 71). The HIGD2A-encoded protein mainly exists in nuclei and mitochondria, and it is essential for the assembly of human mitochondrial complex IV, which can prolong the cell’s lifespan in hypoxic environments. HIGD2A’s expression is markedly elevated in a few cancer tissues, including LUAD, DLBCL, LIHC, and BRCA (72). Additionally, reports have shown that HIGD2A knockdown inhibits the proliferation of HCC cells by interfering with the MAPK/ERK pathway, and persons with lower expression of HIGD2A in LIHC exhibit higher survival rates (73). The HIGD1B genome is located on chromosome 17q21.31. The protein HIG2A encoded by this gene comprises 99 amino acids and is highly expressed in multiple human body tissues. HIGD1B is a crucial collateral homolog of HIGD1A, and they regulate mitochondrial homeostasis in comparable ways. Pang et al. found that HIGD1B promoted cardiomyocyte survival by stabilizing the mitochondrial morphology (16). Additionally, HIGD1B is highly expressed in human lung adenocarcinoma and is associated with a worse outcome (19). However, the role and significance of HIGD1B in gastric cancer have not yet been explored.

In this article, the researchers initially analyzed and corroborated the expression of HIGD1B in queues (TCGA-STAD, GSE54129, and GSE29272) from public databases. Studies implied HIGD1B was significantly upregulated in human GC tissues, as well as qRT-PCR and Western blot, also confirmed that this gene was more expressed in human GC cell lines, suggesting that HIGD1B may have carcinogenic and promoting effects on GC. Next, we downloaded prognostic data for GC patients, and the K-M curve revealed that the OS, PFS, DFS, and DSS of the population with high-HIGD1B expression in the TCGA cohort were shorter (p<0.05). The dependability of this gene in predicting overall survival was verified in the GSE62254 and GSE84437 cohorts, and the ROC curve indicated that HIGD1B may reasonably predict the survival rate of GC patients. In addition, HIGD1B’s expression was elevated in GC patients with G3 grade, later stage, and T stage in clinical groups. Cox regression analysis revealed that age, stage, and HIGD1B expression are independent elements for predicting the outcome of gastric cancer. Subsequently, a nomogram was created using these indicators to predict the outcome of GC patients, and its efficiency and reliability were examined through ROC and calibration curves. Moreover, differential analysis was subjected to the high and low HIGD1B groups, yielding 805 DEGs for enrichment analysis. According to the GSEA results, the “Hedgehog,” “TGF-β,” “MAPK,” and “Wnt” signaling pathways, as well as matrix activation and adhesion, are linked to the HIGD1B high expression group.

In recent years, rapid development in immunotherapy has drawn attention to the tumor microenvironment (TME), which comprises several components, including cells, extracellular matrix, and blood vessels. Among these, immune cells play a dual role in promoting and combating cancer. This study clarified the relationship between HIGD1B, TME, and TIICs and discovered a positive correlation with immune scores and infiltration of tumor-promoting immune cells (such as Tregs, MDSC, and M2 macrophages). It is pertinent to note that indicators connected to immunotherapy are critical in formulating treatment plans for gastric cancer. Consequently, we thoroughly assessed the potential association between HIGD1B and ICIs, TMB, TIDE, and MSS. This research demonstrated that individuals with high HIGD1B had higher TIDE scores and a higher risk of immune evasion. In contrast, persons with low HIGD1B had higher TMB values and MSI-H ratios in gastric cancer and had better efficacy for CTLA-4 immunotherapy. Eventually, drug sensitivity analysis also revealed that the group with low HIGD1B expression exhibited lower IC50 values and better sensitivity to commonly used chemotherapeutic medicines in clinical.

It’s critical to recognize the limitations of this study. Firstly, the data used in the research were all retrieved from public databases, although involving multiple independent queues, there may still be sample bias. Second, although we have validated the differential expression of HIGD1B in gastric cancer cells and normal gastric epithelial cells through partial experiments (qRT-PCR and Western-Blot), further validation in human tissues is still needed. Additionally, more study is required to determine the exact mechanism by which HIGD1B encourages the onset and progression of gastric cancer, and additional experimental data is required to bolster our hypothesis. Finally, this gene has shown considerable potential in predicting immune therapy and clinical outcomes, but it needs to be validated in a clinical cohort of gastric cancer patients. Therefore, in-depth research is crucial for understanding the exact mechanism of this gene.

## 5 Conclusions

This article performed a comprehensive study on the expression pattern and prognostic relevance of HIGD1B in gastric cancer using bioinformatics analysis, elucidated its potential involvement in critical pathways, explored the effects of HIGD1B on the tumor microenvironment (TME) and tumor-infiltrating immune cells (TIICs), and projected the immuno- and chemotherapeutic effects of GC based on HIGD1B expression. In addition, researchers have confirmed the differential expression of HIGD1B in gastric cancer cells and gastric epithelial cells through partial experiments. Thus, there is cause for us to believe that HIGD1B may be a promising biomarker for predicting the outcome of gastric cancer and guiding clinical immunotherapy and personalized treatment.

## Data availability statement

The datasets presented in this study can be found in online repositories. The names of the repository/repositories and accession number(s) can be found in the article/**Supplementary Material**.

## Ethics statement

All data can be obtained from public databases; no ethical approval is required for this study.

## Author contributions

SBW: Data curation, Formal analysis, Methodology, Writing – original draft, Writing – review & editing, Visualization. SYZ: Data curation, Formal analysis, Validation, Writing – original draft, Writing – review & editing. XiaoL: Formal analysis, Methodology, Project administration, Writing – original draft. XianL: Data curation, Software, Validation, Writing – review & editing. SFZ: Formal analysis, Funding acquisition, Writing – review & editing. JG: Formal analysis, Funding acquisition, Writing – review & editing. SSW: Formal analysis, Methodology, Writing – review & editing. RW: Data curation, Visualization, Writing – original draft. MZ: Data curation, Formal analysis, Funding acquisition, Writing – review & editing. WQ: Data curation, Formal analysis, Funding acquisition, Writing – original draft, Writing – review & editing.
